# High flow nasal therapy during early pulmonary rehabilitation in patients with acute severe exacerbation of COPD: beneficial or illusory?

**DOI:** 10.1186/s12931-020-01415-y

**Published:** 2020-06-12

**Authors:** Guillaume Prieur, Yann Combret, Clement Medrinal

**Affiliations:** 1 Institut de Recherche Expérimentale et Clinique (IREC), Pôle de Pneumologie, ORL & Dermatologie, Groupe de Recherche en Kinésithérapie Respiratoire, Université Catholique de Louvain, 1200 Brussels, Belgium; 2 Institute for Research and Innovation in Biomedicine (IRIB), Normandie Univ, UNIROUEN, EA3830-GRHV, 76000 Rouen, France; 3 Groupe Hospitalier du Havre, Pulmonology department and pulmonary rehabilitation department, avenue Pierre Mendes France, 76290 Montivilliers, France

**Keywords:** COPD, Exacerbation, High flow nasal, Pulmonary rehabilitation

## Abstract

In study *“Effect of high-flow nasal therapy during early pulmonary rehabilitation in patients with severe AECOPD: a randomized controlled study”* by Tung et al., authors concluded HFNT utilization led to enhanced exercise tolerance and a reduction of systemic inflammation. Nevertheless, some points requires additional discussion, the conclusion of the trial seems overstated. The baseline differences between groups induces substantial modifications in the conclusions of this trial. HFNT does not seem to add any benefit on exercise tolerance or systemic inflammation, nor on pulmonary function. The only difference that remained significant in homogenous statistical significance is dyspnea on the mMRC scale but clinical significance is highly questionable.

To the Editor,

We read with great interest the study by Tung et al. entitled *“Effect of high-flow nasal therapy during early pulmonary rehabilitation in patients with severe AECOPD: a randomized controlled study”* [1].

In this randomized controlled trial, the authors aimed to evaluate the feasibility and efficacy of high flow nasal therapy (HFNT) during early pulmonary rehabilitation (48 h after hospitalization due to acute exacerbation) in patients with COPD. The authors concluded HFNT utilization led to enhanced exercise tolerance and a reduction of systemic inflammation (C-reactive protein: CRP). Moreover, the authors underlined the hypothesis that HFNT may decrease lung hyperinflation and increase pulmonary function.

Nevertheless, some points requires additional discussion. First, the sample size is small (22 patients per group) and patient allocation resulted in differences in several parameters at baseline (mMRC, CAT and BODE indexes). Patients in the control group presented more severe respiratory symptoms that patients allocated to the HFNT intervention. Rightly, findings were reported as changes from baseline and follow-up at 4 and 12 weeks for these parameters to compare both groups. However, the same strategy was not applied to report the findings of all the outcomes measured in this study.

The study findings reported an increase in exercise tolerance associated with the use of HFNT during early pulmonary rehabilitation. For the analysis of exercise tolerance, baseline comparisons revealed a questioning trend (*p* = 0.052) that favored the exercise capacity in the HFNT group with a mean difference of 48.5 m. Considering this large difference, the same strategy that the one applied for mMRC, CAT and BODE indexes (e.g. comparisons of delta rather that absolute values) should have been applied as well. The within-group change indicate that the 6-min walking distance increased similarly in the HFNT and in the control group (mean increase 125.7 m and 117.0 m, respectively). In the present case, the mean difference between groups would have finally been an increase of 8.7 m in favor of the HFNT group that would not have reached statistical significance.

A significant decrease in dyspnea (measured by the mMRC score) was also reported for the HFNT group at 12 weeks of follow-up by comparing the mean changes from baseline in both groups (mean difference 0.27 points (CI 95% 0.061 to 0.48)). Although the statistical analysis of this outcome reached significance, the difference and CI reported was lower that the MCID of 0.6 points chosen by the authors (the reference cited by authors is not correct). The clinical significance of this result could therefore deserve further discussion.

Furthermore, the authors stated that *“Serum CRP may provide prognostic information about morbidity and mortality in COPD [ …*]. *Our study proved that an HFNT PR program reduced CRP levels better than a non-HFNT PR program”*. It is important to note that CRP levels at 12 weeks were very low for both HFNT and control groups leading to very limited clinical relevance (0.07 ± 0.12 mg/dl and 0.30 ± 0.39 mg/dl, respectively). The previously reported statement that HFNT reduces systemic inflammation therefore seems confusing. Patients in the control group presented higher CRP levels at baseline that nearly reached statistical significance (*p* = 0.086). Alike analysis of exercise tolerance, delta CRP levels (baseline - 12 weeks) should have been compared between groups. Besides, between groups differences in change from baseline seemed to highlight a significant higher diminution in the control group compared to the HFNT group (mean decrease − 8.1 mg/dl and − 5.04 mg/dl, respectively).

To conclude, after a careful examination of the results presented in this study, the conclusion of the trial seems overstated. We believe that the authors should have undertaken homogenous statistical analysis for all the variables evaluated in the present study (as inspiratory capacity, mMRC scale, CAT and BODE index). The baseline differences between groups induces substantial modifications in the conclusions of this trial. Given the points mentioned above, HFNT does not seem to add any benefit on exercise tolerance or systemic inflammation, nor on pulmonary function. The only difference that remained significant in homogenous statistical significance is dyspnea on the mMRC scale but clinical significance is highly questionable. These points raise some concerns that should be further discussed for transparency, especially since the addition of HFNT during exercise seems to induce substantial patient discomfort [2].

## Data Availability

Not applicable.

## Abbreviations

AECOPD: Acute exacerbation of COPD
BODE: health index: Body-mass index, degree of airway obstruction and dyspnea, and exercise capacity
CAT: COPD assessment test
CI: Confidence interval
COPD: Chronic obstructive pulmonary disease
CRP: C-reactive protein
HFNT: High flow nasal therapy
MCID: Minimal clinically important difference
mMRC: Modified medical research council scale
